# Calcium dominance, ion regulation, and metabolic defenses underlie salt tolerance in the halophyte *Azima sarmentosa*

**DOI:** 10.7717/peerj.21162

**Published:** 2026-04-21

**Authors:** Piyanut Khanema, Woraporn Laojinda, Catleya Rojviriya, Jinnawat Manasathien

**Affiliations:** 1 Department of Biology, Faculty of Science, Mahasarakham University, Maha Sarakham, Thailand; 2 Isan Saline Soil Research Unit (ISSRU), Faculty of Science, Mahasarakham University, Maha Sarakham, Thailand; 3 Faculty of Science, Mahasarakham University, Maha Sarakham, Thailand; 4 Synchrotron Light Research Institute (Public Organization), Nakhon Ratchasima, Thailand; 5 Faculty of Science and Technology, Nakhon Ratchasima Rajabhat University, Nakhon Ratchasima, Thailand

**Keywords:** Antioxidant defense, *Azima sarmentosa*, Biomineralization, Calcium oxalate, Halophyte, Saline–sodic soil, Salt tolerance, Salvadoraceae

## Abstract

**Background:**

Soil salinity is a major constraint to plant productivity, yet halophytes have evolved diverse strategies to tolerate excess salt. *Azima sarmentosa*, a woody halophyte native to Southeast Asia, thrives in saline, calcium-poor soils, but its underlying tolerance mechanisms remain insufficiently described. This study investigated anatomical, physiological, and biochemical traits associated with salinity tolerance across a natural salinity gradient.

**Methods:**

Soils, stems, mature leaves, and young leaves were analyzed using ion quantification, scanning electron microscopy Energy Dispersive X-ray Spectrometer (SEM-EDS/EDX), synchrotron radiation X-ray tomographic microscopy (SRXTM), Fourier transform infrared spectroscopy (FT-IR) spectroscopy, and multivariate analyses (correlation and Principal Component Analysis (PCA)) to characterize Ca–, Na–, and metabolic-related responses.

**Results:**

Despite low soil Ca^2+^, plants maintained high Ca^2+^/Na^+^ ratios and produced abundant Ca-oxalate (CaOx) crystals in leaves and stems, indicating selective calcium uptake and biomineralization. SEM–EDS/EDX confirmed Ca-rich deposits and bicellular salt glands on both leaf surfaces, while SRXTM visualized their three-dimensional distribution within tissues. Young leaves accumulated high levels of proline, phenolics, and flavonoids, supporting osmotic adjustment and antioxidant protection. FT-IR spectra corroborated the presence of phenolic functional groups. Correlation analysis and PCA revealed a strong antagonism in trait associations: Ca-related variables clustered with pigments, proline, flavonoids, whereas Na^+^/Cl^−^ grouped with EtOH-derived phenolics, highlighting a divergence between Ca-driven protection and Na-linked stress.

**Conclusion:**

*A. sarmentosa* withstands salinity through an integrative, calcium-centered strategy involving selective Ca^2+^ uptake, CaOx biomineralization, salt secretion, and metabolic defenses. Unlike halophytes that rely mainly on sodium sequestration, this species exhibits a distinctive Ca-based adaptation. CaOx formation not only immobilizes Ca^2+^ but also incorporates CO_2_-derived oxalate, linking ionic regulation with carbon cycling and broadening the ecological significance of calcium-mediated salt tolerance.

## Introduction

Soil salinity is one of the most critical abiotic stresses limiting agricultural productivity and ecosystem stability worldwide. It already affects more than 20% of irrigated lands and is projected to threaten even larger areas in the future (Rahman et al., 2021). High salt concentrations reduce plant growth by inducing osmotic stress, ionic toxicity, nutrient imbalance, and oxidative damage, which collectively impair photosynthesis and metabolic functioning (Rahman et al., 2021; Garcia-Caparros et al., 2022; Hasanuzzaman & Fujita, 2022). Halophytes have therefore received increasing attention not only for their tolerance mechanisms but also for their potential application in saline agriculture (Ventura & Sagi, 2013), especially as global salinization continues to expand.

Halophytes, which represent less than 2% of terrestrial plant species, possess a wide range of intrinsic strategies that enable survival in saline and sodic soils (Shabala, 2013; Patel et al., 2020). These strategies include selective ion uptake or exclusion, vacuolar sequestration of Na^+^ and Cl^−^, salt secretion through salt glands or bladder cells, osmotic adjustment *via* compatible solutes such as proline and sugars, and the activation of antioxidant defense systems (Tan et al., 2013; Rahman et al., 2021; Liu et al., 2024). Recent studies illustrate the complexity of ion regulation in halophytes; for example, *Salicornia fruticosa* exhibits tissue-specific partitioning of Na^+^, K^+^, and Ca^2^^+^ as well as differential osmolyte distribution under varying salinity levels (Marco, Carvajal & Carmen Martinez-Ballesta, 2019). Other halophytes, such as *Salicornia brachiata*, have been shown to improve soil quality by reducing exchangeable Na^+^ and enhancing nutrient availability, indicating their potential role in rehabilitating degraded saline soils (Rathore, Chaudhary & Jha, 2022). Together, these examples highlight the ecological and functional diversity of halophytes and provide valuable comparative context for understanding salt tolerance strategies in other species.

Calcium also plays a central role in maintaining ionic balance, stabilizing membranes, and mediating stress signaling (Hadi & Karimi, 2012; Zhou et al., 2024). Moreover, the formation of calcium oxalate (CaOx) crystals is considered to be an important mechanism for reducing cytotoxic Ca^2^^+^ concentrations while contributing to structural reinforcement and potential roles in metabolic buffering (Cuadra & Cambi, 2017; De la Fuente et al., 2018). Recent anatomical studies have shown that CaOx crystals may occur in close association with chloroplast structures and carbon metabolism, suggesting their possible involvement in photosynthetic adjustment under stress (Cerritos-Castro et al., 2022).

*Azima sarmentosa* (Blume) Benth. & Hook.f., a member of the Salvadoraceae, is distributed across South and Southeast Asia. In Northeast Thailand, where saline soils are widespread, this species commonly occurs on salt-affected sites and is capable of persisting under high soil salinity conditions. Previous phytochemical studies have identified various secondary metabolites and compounds with antioxidant activity in this species (Sunintaboon, 1999; Poodeetip et al., 2013). However, despite these biochemical evaluations, the anatomical and integrative physiological bases of its salt tolerance remain poorly understood.

Because salt-tolerance mechanisms differ among plant organs, we compared stems, mature leaves, and young leaves to capture tissue-specific responses. Specifically, we investigated (i) elemental ion accumulation and localization using scanning electron microscopy (SEM), scanning electron microscopy Energy Dispersive X-ray Spectrometer (SEM-EDS/EDX), and synchrotron radiation X-ray tomographic microscopy (SRXTM); (ii) osmoprotectant and antioxidant contents across tissues; and (iii) functional groups in crude extracts using Fourier transform infrared spectroscopy (FT-IR) spectroscopy. These integrated analyses aim to clarify the potential Ca-centered adaptations, as well as other mechanisms, supporting the salt tolerance of *A. sarmentosa*.

## Materials and Methods

### Study site and plant collection

**Figure 1:**
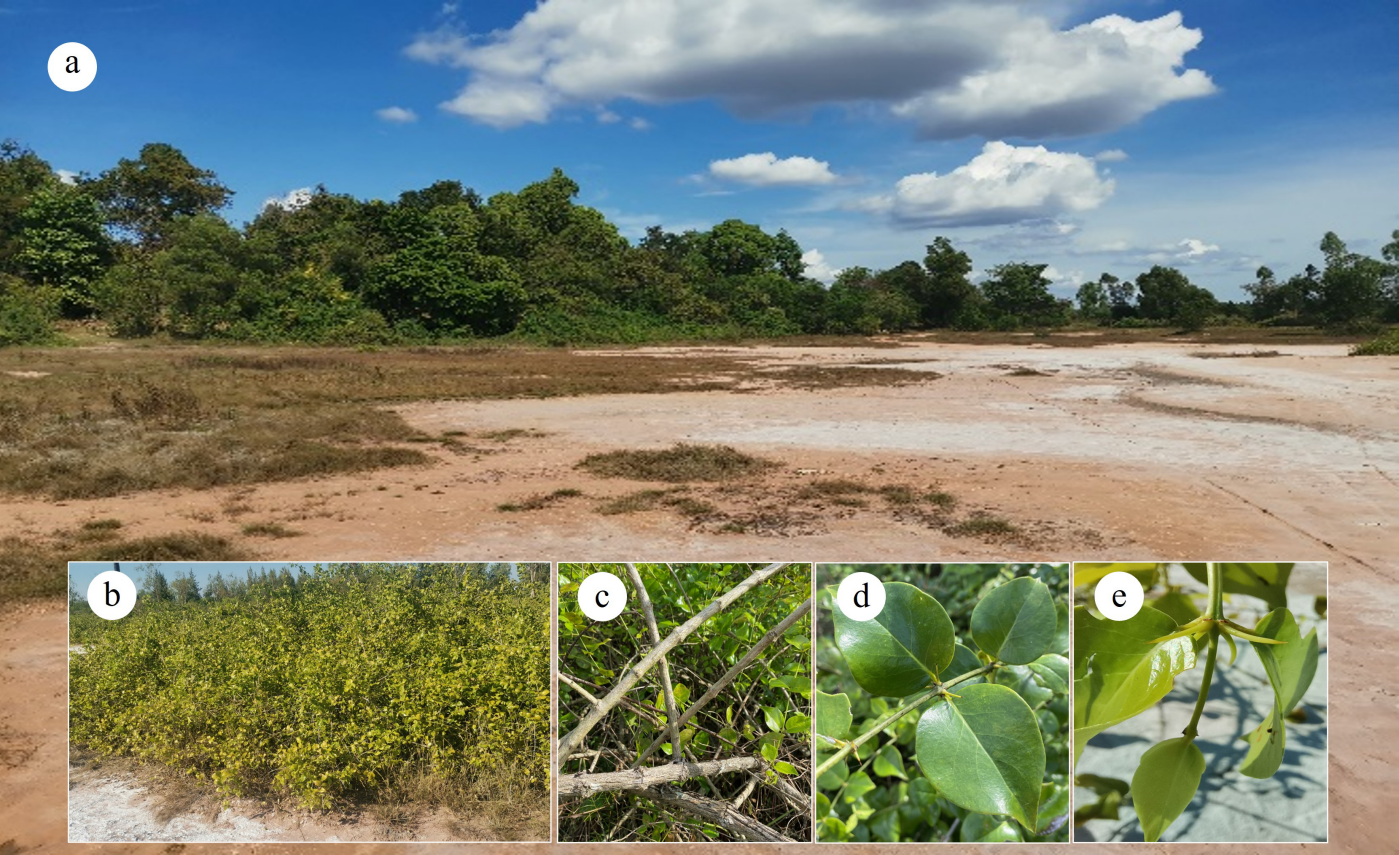
Habitat and morphological characteristics of *Azima sarmentosa*. (A) Saline field site in Kalasin Province, Thailand, showing visible salt crust and halophytic vegetation. (B) Representative *A. sarmentosa* individual. Detailed morphological structures include (C) stems with woody and spiny architecture, (D) mature leaves, and (E) young leaves.

*Azima sarmentosa* specimens were collected in April 2024 from a naturally saline site with a history of traditional salt boiling in Khlong Kham Sub-district, Yang Talat District, Kalasin Province, Thailand (16°24′20.0″N, 103°17′07.7″E) (Fig. 1A). The area features exposed saline crusts, sandy-loam soil, and sparse halophytic vegetation.

We used a shrub-centered stratified random design reflecting the species’ patchy distribution (Fig. 1B). Five healthy clumps were selected as biological replicates; from each, stems, mature leaves, and young leaves were sampled to capture tissue-level variation (Figs. 1C–1E). Samples were transported to the laboratory for anatomical, biochemical, and elemental analyses.

### Soil collection and preparation

Soil samples were collected from two depth intervals—topsoil (0–50 cm) and subsoil (51–100 cm)—beneath each *A. sarmentosa* clump. For each depth layer, 15 soil cores were randomly collected within a 1-meter radius of the clump base. The cores were thoroughly mixed to form a composite sample per depth. A quartering method was used to subsample approximately one kg of soil for laboratory analysis. This resulted in two composite samples per clump, corresponding to the two soil layers.

### Soil physicochemical analysis

Exchangeable cations (Ex-K^+^, Ex-Na^+^, Ex-Ca^2^^+^, Ex-Mg^2^^+^) were extracted with 1 N ammonium acetate (pH 7.0), quantified using atomic absorption spectroscopy (AAS; Agilent 280FS AA), and expressed as cmolc kg^−1^, following the protocol of the (Land Development Department, 2010). Chloride (Cl^−^) was determined by Mohr titration (Sheen & Kahler, 1938) and reported as mg kg^−1^. Sodium adsorption ratio (SAR) was calculated as follows:

\begin{eqnarray*}SAR= \frac{N{a}^{+}}{\sqrt{ \left( \frac{C{a}^{2+}+M{g}^{2+}}{2} \right) }} \end{eqnarray*}

where all ion concentrations are expressed in cmolc kg^−1^. Soil pH was measured in a 1:5 soil-to-water suspension using a calibrated pH meter. Electrical conductivity of the saturation extract (EC_e_) was measured with a conductivity meter. Soil organic carbon (SOC) was analyzed by the Walkley–Black method (Walkley & Black, 1934), and total organic nitrogen (TON) by Kjeldahl digestion (Bremner, 1960).

### Elemental accumulation in plant tissues

To determine elemental accumulation in relation to salinity, dried plant tissues were divided into stems, mature leaves, and young leaves. Samples were digested using a mixture of nitric and perchloric acids (HNO_3_:HClO_4_), following the guidelines of Association of Official Analytical Chemists Official Method 975.03 (Isaac, 1990). The concentrations of calcium (Ca), sodium (Na), potassium (K), and magnesium (Mg) were measured using AAS, with results reported on a dry weight basis (g kg^−1^ DW).

### Scanning electron microscopy coupled with energy-dispersive X-ray spectroscopy

Transverse and radial sections of stems, mature leaves, and young leaves were fixed, dehydrated, and sputter-coated with gold prior to imaging. SEM was performed using a HITACHI TM4000 Plus (Hitachi High-Tech Corporation, Tokyo, Japan) to examine tissue morphology and crystalline deposits. Elemental composition was analyzed by energy-dispersive X-ray spectroscopy (EDS/EDX) coupled to the SEM. SEM–EDX data are reported as weight % (normalized mass concentration). For each tissue type (two stems, two mature leaves, one young leaf), five regions per specimen were scanned (totaling 25 EDX spectra).

### Synchrotron radiation X-ray tomographic microscopy

SRXTM was performed on leaf and stem segments using a filtered polychromatic beam with a mean photon energy of approximately 11 keV at Beamline 1.2W of the Siam Photon Source, Synchrotron Light Research Institute (Thailand). All X-ray images were obtained with an isotropic voxel size of 3.61 µm using an imaging system consisting of a YAG:Ce scintillator, a 2X objective lens coupled with a microscope (Optique Peter, France), and an Andor Neo 5.5 sCMOS camera (Oxford Instrument, Abington, Oxfordshire). Each tomography scan consisted of 601 projections over 180°, with 0.3° increments, and included bright-field and dark-field images for flat-field correction. Multiple scans were conducted to cover entire leaf and stem samples, incorporating a 10% overlap between adjacent fields of view. The filtered back-projection algorithm in Octopus Reconstruction (Inside Matters, Belgium) was used for reconstruction, and 3D rendering and visualization were completed in Drishti (Limaye, 2012).

### Determination of photosynthetic pigments

Chlorophyll and total carotenoids were quantified according to (Costache, Campeanu & Neata, 2012) by extracting fresh samples in acetone (1:50, w/v) and recording absorbance at 470, 645, and 662 nm.

### Determination of proline and total soluble sugars

Proline was extracted in 3% sulfosalicylic acid and quantified by the acid-ninhydrin method (Bates, Waldren & Teare, 1973), with absorbance at 520 nm against an L-proline standard curve. Total soluble sugars were extracted in 80% ethanol and determined by the phenol–sulfuric acid method (Dubois et al., 1956), using glucose as the standard and absorbance at 490 nm. Values were normalized to dry weight (DW) using subsamples oven-dried at 70 °C.

### Extraction and quantification of phenolics and flavonoids

Powdered tissues were extracted with acetone or ethanol (1:10, w/v) for 48 h using Soxhlet, concentrated, lyophilized, and stored at −20 °C. Total phenolics and flavonoids were determined following Marinova, Ribarova & Atanassova (2005): phenolics by the Folin–Ciocalteu method with absorbance at 750 nm, expressed as mg gallic acid equivalent (GAE) g^−1^ DW; flavonoids by the aluminum chloride assay with absorbance at 510 nm, expressed as mg quercetin equivalent (QE) g^−1^ DW.

### Phenolic and flavonoid profiling *via* FT-IR spectroscopy

Acetone and ethanol extracts obtained from the phenolic and flavonoid determination procedure were re-dissolved in their respective solvents and analyzed using a Bruker Invenio-S FT-IR spectrometer (Bruker, Germany) equipped with an ATR diamond crystal. Each sample was air-dried on the crystal to remove residual solvent prior to measurement. Spectra were recorded in MIR-ATR mode across 4,000–400 cm^−1^ at a resolution of 4 cm^−1^, with 32 scans collected per sample. Characteristic absorption bands were identified to assign functional groups associated with phenolic and flavonoid compounds.

### Statistical analysis

All traits were assessed using five biological replicates; triplicate technical measurements were averaged to yield one value per biological replicate. Data are presented as means ±  SD (*n* = 5). Differences in soil properties between topsoil and subsoil were tested with paired-sample *t*-tests. One-way and two-way ANOVA with Tukey’s HSD (*p* < 0.05) were used to assess elemental concentrations, photosynthetic pigments, osmoprotectants, and antioxidant compounds. Pearson’s correlation examined pairwise relationships, while Principal Component Analysis (PCA) with Varimax rotation and Kaiser normalization was used to explore multivariate trait associations.

## Results

### Soil properties of the study site

Soils at the study site were classified as saline–sodic, with EC_*e*_ values exceeding 17 dS m^−1^ and SAR averaging ∼16 (Table 1). Exchangeable Na^+^ was the dominant cation, whereas Ex-Ca^2^^+^ and Ex-Mg^2^^+^ were extremely low (<0.5 cmolc kg^−1^). Chloride concentrations were relatively high, and soil pH was moderately acidic (∼5.0). No significant differences were detected between topsoil (0–50 cm) and subsoil (51–100 cm), indicating consistently sodium-dominated and calcium-deficient conditions throughout the soil profile.

**Table 1:** Soil properties of the study site at two depths (0–50 cm and 51–100 cm).

Soil property	Soil depth		*t*-test	Significant
	0–50 cm	51–100 cm		
Ex-K^+^ (cmolc kg^−1^)	0.09 ± 0.05^a^	0.08 ± 0.03	1.32	0.26^ns^
Ex-Na^+^ (cmolc kg^−1^)	9.58 ± 3.73	7.79 ± 2.07	2.11	0.10^ns^
Ex-Ca^2+^ (cmolc kg^−1^)	0.43 ± 0.19	0.33 ± 0.11	1.75	0.15^ns^
Ex-Mg^2+^ (cmolc kg^−1^)	0.31 ± 0.23	0.18 ± 0.06	1.61	0.18^ns^
Cl^−^ (g kg^−1^)	2.39 ± 0.87	1.86 ± 0.53	2.27	0.09^ns^
SAR	16.12 ± 4.65	15.78 ± 4.21	0.30	0.78^ns^
EC_e_ (dS m^−1^)	21.79 ± 7.54	17.81 ± 4.98	1.74	0.16^ns^
pH	5.29 ± 0.58	5.08 ± 0.68	1.02	0.36^ns^
SOC (%)	12.96 ± 5.98	14.34 ± 5.69	−1.08	0.34^ns^
TON (%)	0.65 ± 0.30	0.72 ± 0.28	−1.09	0.34^ns^

**Notes.**

a Data are presented as means ± SD (*n* = 5).

“ns” indicates no significant difference. Abbreviations: Ex, exchangeable; SAR, sodium adsorption ratio; EC_e_, electrical conductivity of saturation extract; SOC, soil organic carbon; TON, total organic nitrogen.

### Salt element accumulation in plant tissues

**Figure 2:**
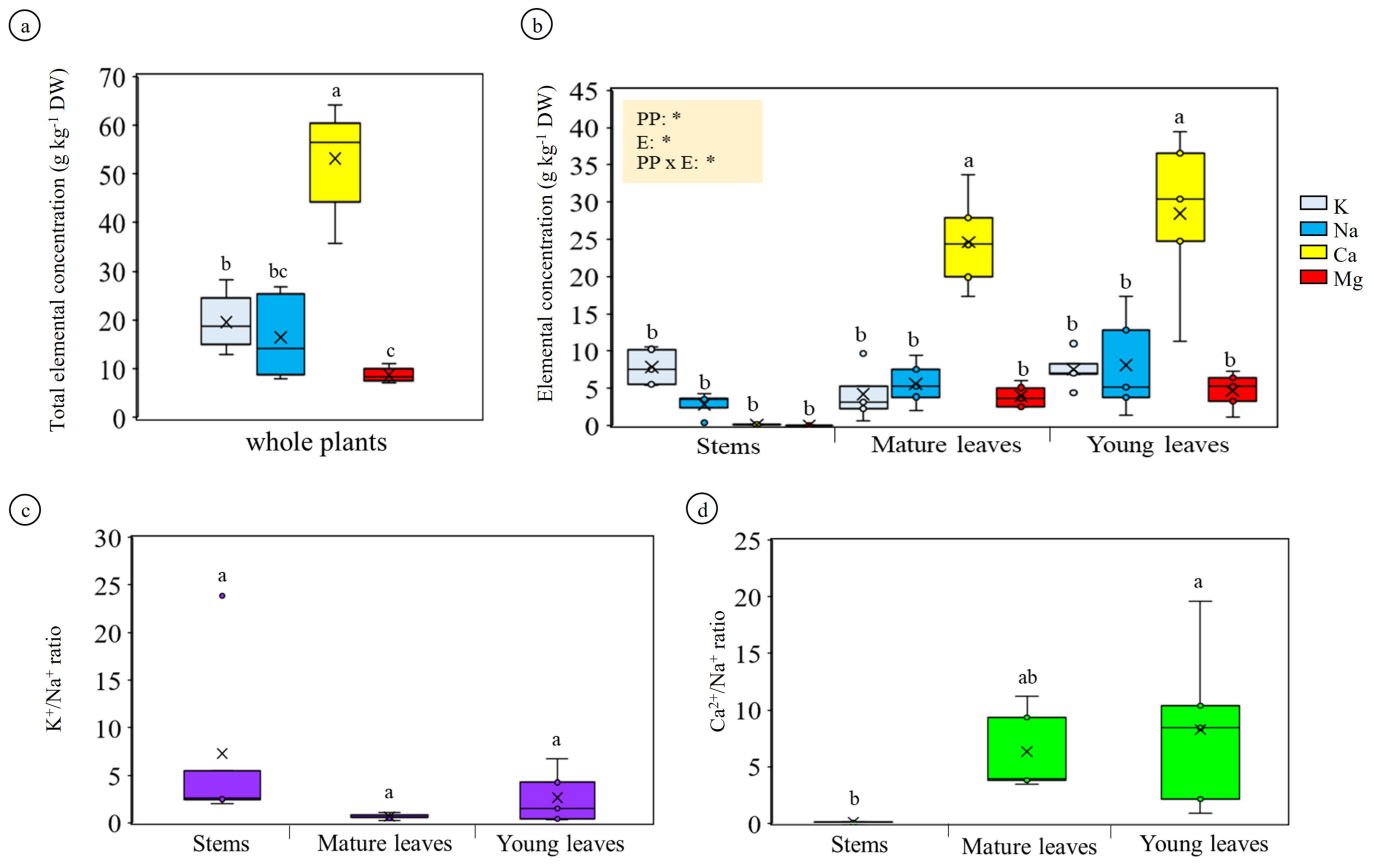
Accumulation and distribution of salt-related elements in *Azima sarmentosa*. (A) Total concentrations of K, Na, Ca, and Mg in whole plants. (B) Elemental concentrations in stems, mature leaves, and young leaves. Inset shows two-way ANOVA significance for plant part (PP), element (E), and interaction (PP × E). Letters above bars indicate significant differences among PP × E combinations (Tukey HSD). (C) K^+^/Na^+^ and (D) Ca^2^ +/Na^+^ ratios across plant parts. Data are means ± SD (*n* = 5).

AAS analysis showed that Ca was the most abundant cation in *A. sarmentosa* (∼53 g kg^−1^ DW), followed by Na and K, with Mg being lowest (Fig. 2A). At the tissue level, Ca was strongly enriched in leaves—especially young leaves—while Na was moderate in leaves and lowest in stems; K remained relatively uniform among tissues (Fig. 2B). The K^+^/Na^+^ ratio did not differ significantly among tissues (Fig. 2C), whereas the Ca^2^^+^/Na^+^ ratio was highest in young leaves and lowest in stems (Fig. 2D), demonstrating preferential Ca accumulation in developing tissues.

### Crystalline ion accumulation and elemental composition in plant tissues

**Figure 3:**
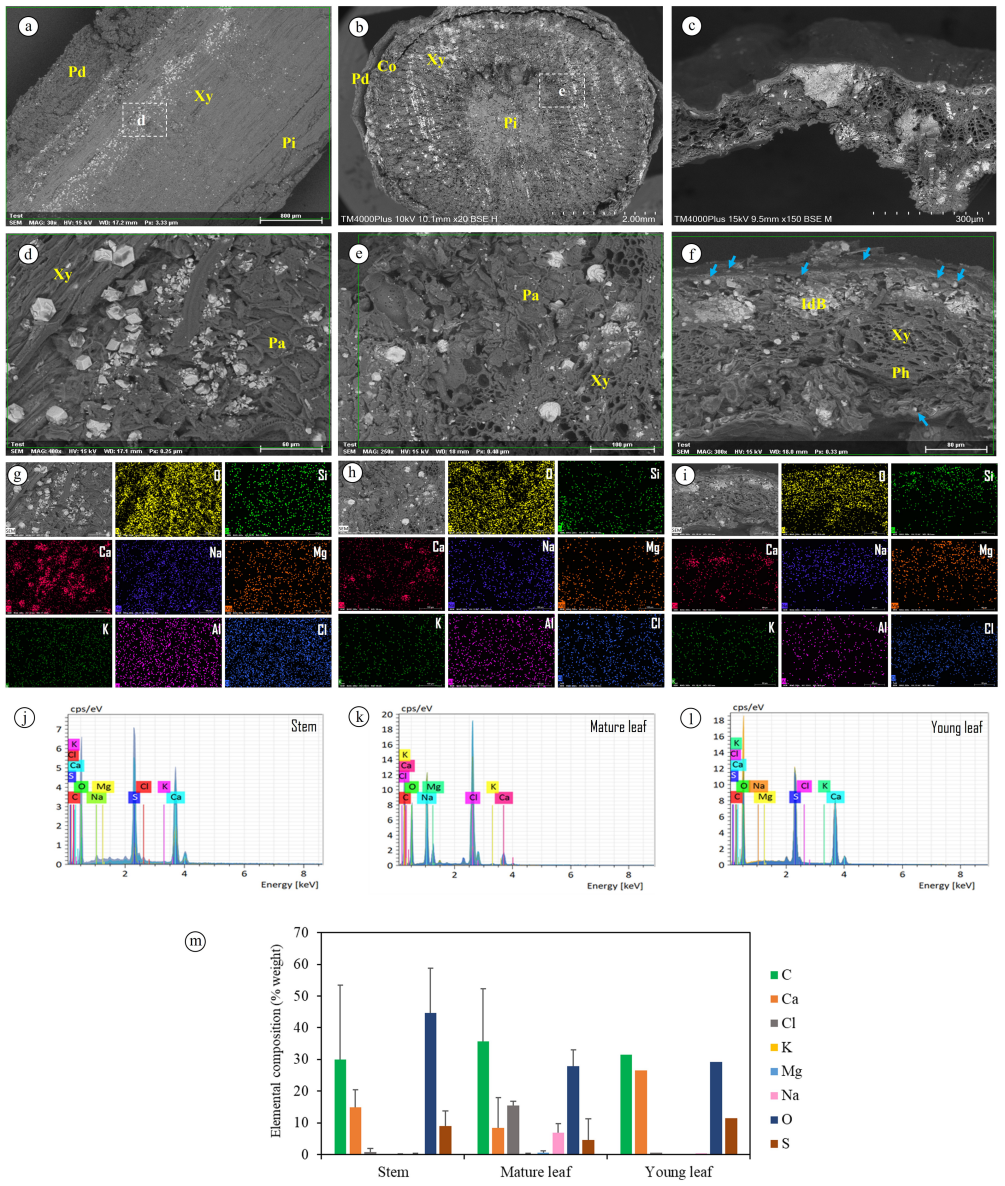
SEM–EDS–EDX analysis of *Azima sarmentosa* tissues. (A) SEM tangential section of stem, (B) stem cross-section, and (C) leaf cross-section showing crystalline deposits in periderm (Pd), cortex (Co), xylem (Xy), pith (Pi), parenchyma (Pa), and idioblasts (IdB). (D–F) Higher-magnification views highlighting crystalline accumulations; blue arrows in (F) mark putative salt glands near the epidermis, while phloem (Ph) is labeled for reference. (G–I) SEM–EDS elemental maps showing distributions of O, Si, Ca, Na, Mg, K, Al, and Cl. (J–L) SEM–EDX spectra from stem, mature leaf, and young leaf. (M) Semi-quantitative comparison of mean normalized mass concentrations (% weight) across tissues, averaged from 25 spectra (five scans × five specimens). Error bars indicate SD among biological replicates (*n* = 2 for stems and mature leaves, *n* = 1 for young leaf).

SEM revealed abundant crystalline deposits in stems and leaves, particularly in the periderm, cortex, xylem, parenchyma, and idioblasts (Figs. 3A– 3F). Putative salt glands were also observed on both epidermal surfaces (Fig. 3F), indicating an external route of ion excretion. Elemental maps (SEM–EDS) showed that Ca and O dominated the crystalline deposits, accompanied by detectable signals of Si, Na, Mg, K, Al, and Cl (Figs. 3G–3I). Point spectra (SEM–EDX) further confirmed Ca-rich profiles in stems and young leaves, whereas mature leaves contained additional Na and Cl (Figs. 3J–3M).

### SRXTM reveals 3D distribution of internal crystals

**Figure 4:**
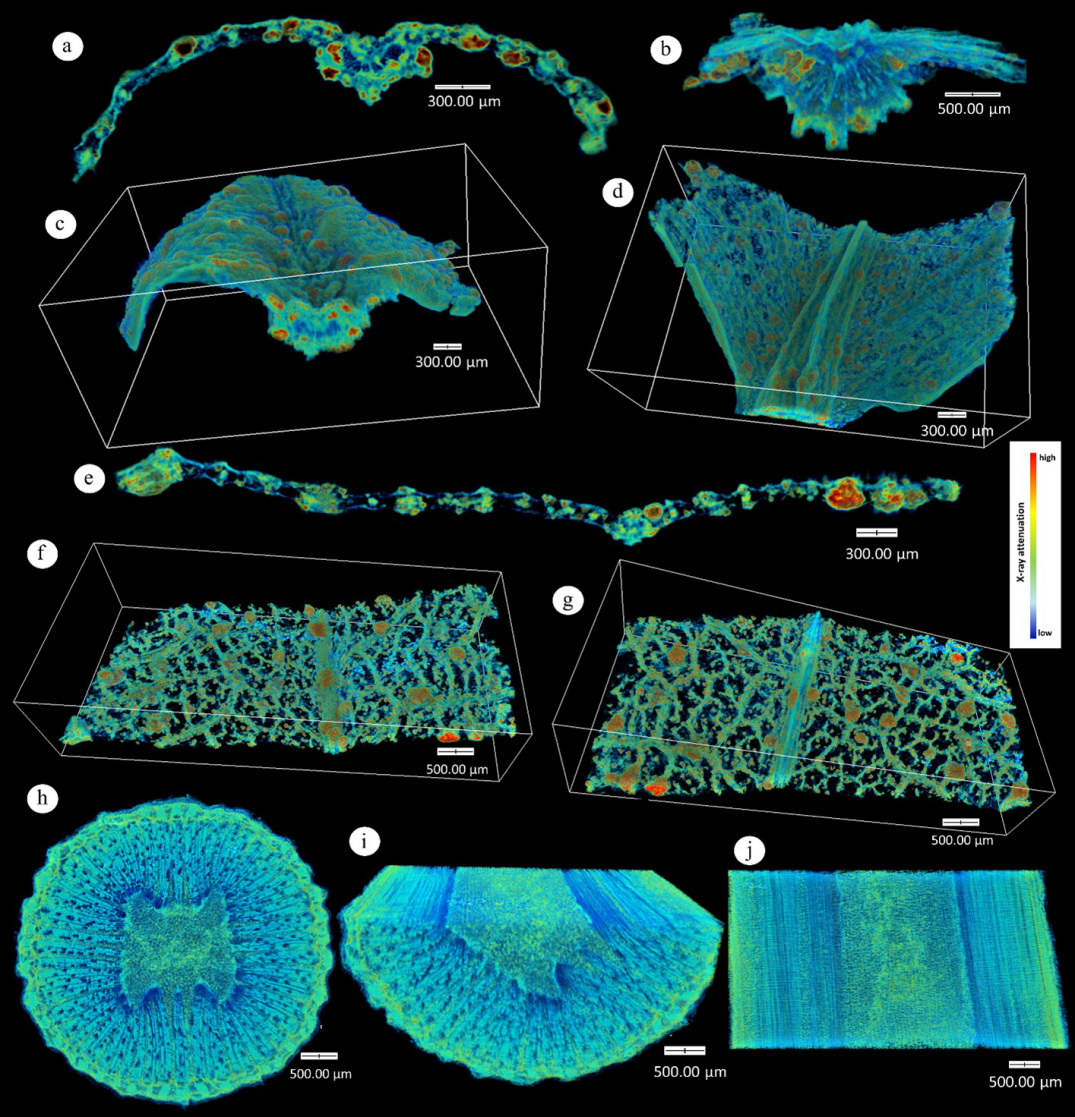
Three-dimensional X-ray tomography (XTM) of *Azima sarmentosa* tissues. (A–D) Young leaves: (A) transverse section of leaf blade; (B) cross-section of midrib; (C) adaxial tissue; (D) abaxial tissue. (E–G) Mature leaves: (E) transverse section; (F) adaxial region; (G) abaxial region. (H–J) Stem: (H) transverse section; (I) cross-section of central tissues; (J) tangential view. Regions of high X-ray attenuation (orange-red) represent dense crystalline or ion-rich deposits.

SRXTM imaging provided three-dimensional visualization of dense particles within *A. sarmentosa* tissues (Fig. 4). In young leaves, crystals were concentrated along both epidermal layers and around vascular tissues, with localized zones of high attenuation in the midrib. Mature leaves exhibited more widespread mesophyll deposits that remained most prominent near vascular bundles and epidermal regions. In stems, dense particles formed radial patterns along the vascular cylinder and pith. Higher brightness (red–orange) indicates regions of elevated X-ray attenuation corresponding to crystalline or ion-rich aggregates (*e.g.*, Ca^2^^+^, Na^+^), highlighting their significance in internal ion sequestration.

### Osmoprotectant and antioxidant responses across plant tissues

**Figure 5:**
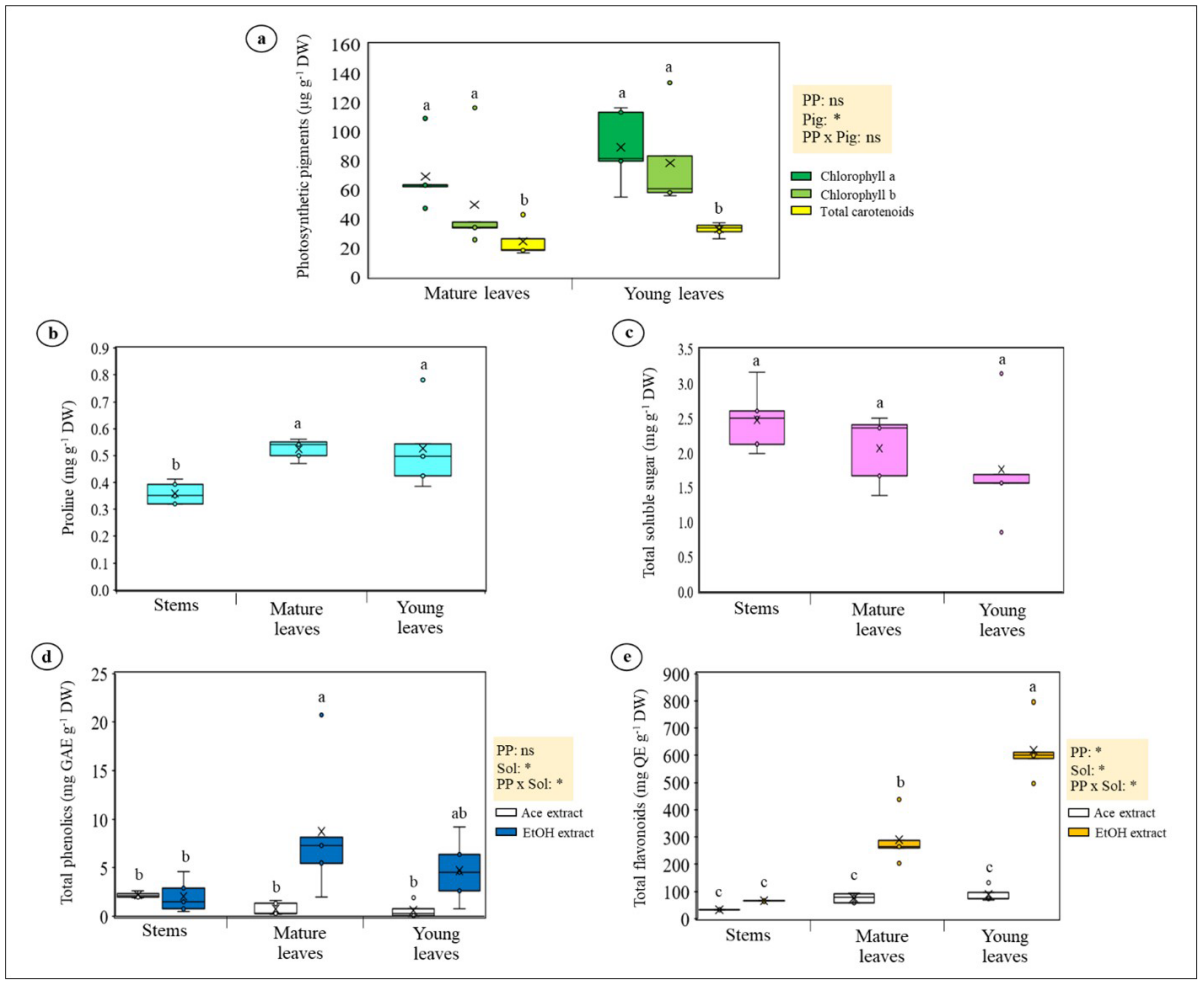
Photosynthetic pigments, osmoprotectants, and antioxidant compounds in *Azima sarmentosa*. (A) Photosynthetic pigments (chlorophyll a, chlorophyll b, total carotenoids); (B) proline; (C) total soluble sugars; (D) total phenolics; and (E) total flavonoids measured in acetone (Ace) and ethanol (EtOH) extracts. Data expressed on dry-weight basis. Statistical effects from ANOVA for plant part (PP), pigment type (Pig), solvent (Sol), and interactions (PP × Pig, PP × Sol) are shown. Asterisks (*) denote significant effects; “ns” = not significant. Different letters indicate significant group differences. Means ± SD (*n* = 5).

Photosynthetic pigment concentrations were similar between mature and young leaves (Fig. 5A). Proline showed strong tissue variation, being highest in young leaves and lowest in stems (Fig. 5B). Soluble sugars were elevated in stems and mature leaves but lower in young leaves (Fig. 5C). Total phenolics were higher in ethanol than acetone extracts, with mature leaves exceeding stems (Fig. 5D). Flavonoids showed the clearest tissue-specific pattern: young leaves accumulated the highest levels, followed by mature leaves and stems (Fig. 5E). These patterns collectively indicate that young leaves served as the primary sites of osmotic adjustment and antioxidant activity.

### Functional group analysis of extracts using FT-IR spectroscopy

**Figure 6:**
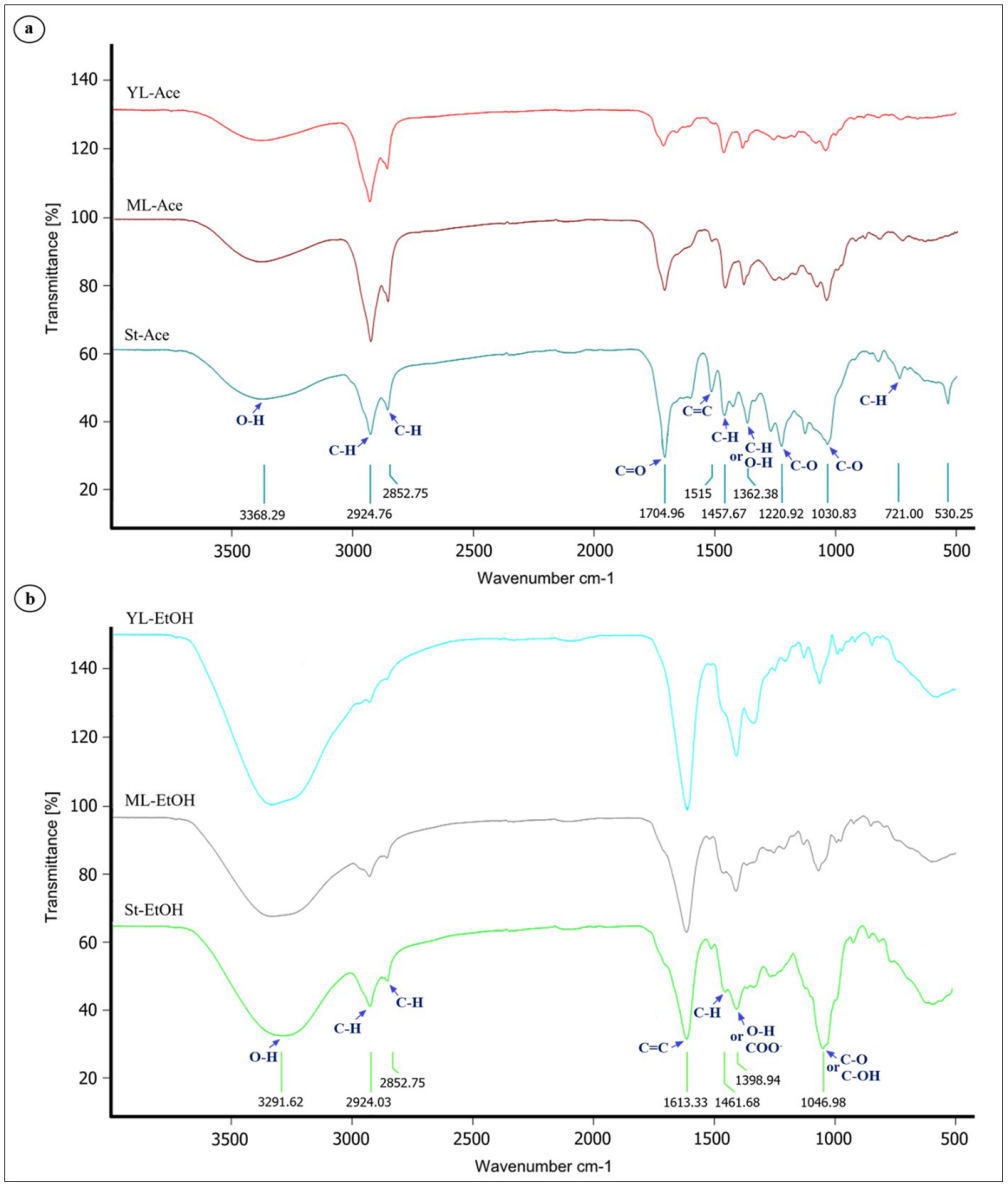
FT-IR spectra of *Azima sarmentosa* crude extracts. (A) Acetone (Ace) and (B) ethanol (EtOH) extracts from stems (St), mature leaves (ML), and young leaves (YL). Key absorption bands: O–H stretch (∼3,368–3,291 cm^−1^), C–H stretch (∼2,924 and 2,853 cm^−1^), aromatic C=C (∼1,613 cm^−1^ in EtOH extracts), phenolic O–H deformation/COO− stretch (∼1,399 cm^−1^), and C—O stretch (∼1,220–1,047 cm^−1^).

FT-IR analysis identified characteristic functional groups associated with phenolics, flavonoids, and related metabolites (Fig. 6). Both acetone and ethanol extracts showed broad O–H stretching bands near 3,360–3,290 cm^−1^, consistent with hydroxyl groups. Peaks at ∼2,924 and 2,853 cm^−1^ represented aliphatic C–H stretching. In the fingerprint region, ethanol extracts exhibited a distinct band around 1,613 cm^−1^ corresponding to aromatic C=C stretching (Fig. 6B), whereas acetone extracts displayed a broader feature between 1,700–1,610 cm^−1^, indicative of overlapping C=O or C=C signals (Fig. 6A). Additional bands at 1,462–1,399 cm^−1^ reflected C–H bending, O–H deformation, and/or COO^−^ symmetric stretching. Strong C–O stretching peaks at 1,220–1,047 cm^−1^ were characteristic of alcohols, phenols, or glycosidic linkages. Peak intensities varied with tissue type and solvent, with young leaf ethanol extracts showing the strongest phenolic-associated absorbance.

### Correlation and principal component analysis of physiological, biochemical, and elemental traits

**Figure 7:**
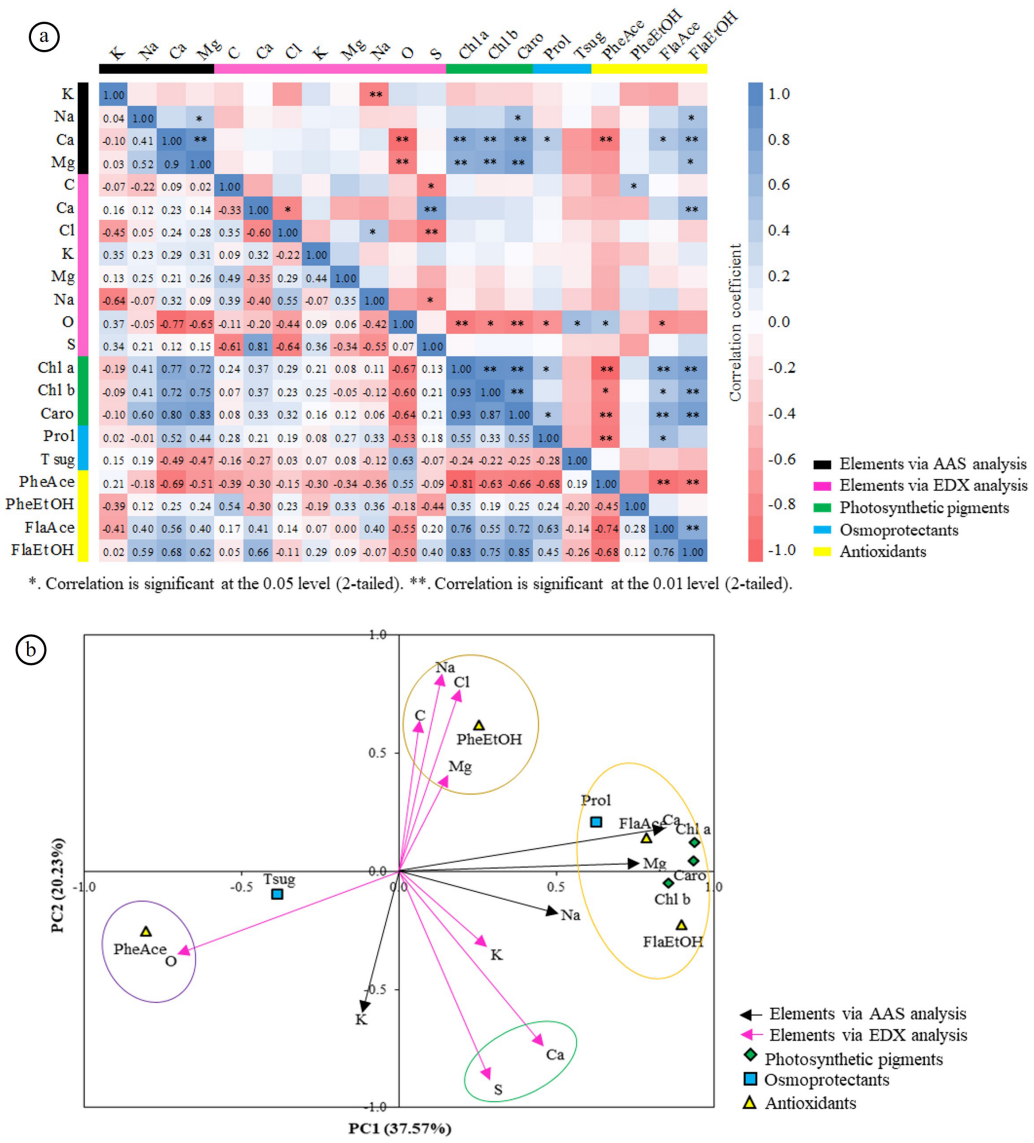
Correlation and principal component analysis (PCA) of physiological and elemental traits in *Azima sarmentosa*. (A) Pearson correlation heatmap among elemental concentrations (AAS, EDX), photosynthetic pigments, osmoprotectants, and antioxidants. Positive and negative correlations shown in blue and red; ^∗^*p* < 0.05, ^∗∗^*p* < 0.01. Trait groups: black (AAS elements), pink (EDX elements), green (photosynthetic pigments), turquoise (osmoprotectants), yellow (antioxidants). (B) PCA biplot along PC1 and PC2; colored ellipses group traits with similar loadings. Abbreviations: Prol = proline; Tsug = total soluble sugars; PheAce/EtOH = phenolics (acetone/ethanol); FlaAce/EtOH = flavonoids (acetone/ethanol); Chl a/b = chlorophyll a/b; Caro = carotenoids.

Correlation analysis revealed that Ca (AAS) was strongly and positively associated with Mg, photosynthetic pigments, proline, and flavonoids, while negatively correlated with oxygen and acetone-derived phenolics (PheAce) (Fig. 7A). Ca (EDX) correlated positively with S and ethanol-derived flavonoids (FlaEtOH) but negatively with Cl. Na (AAS) showed positive relationships with Mg, carotenoids, and FlaEtOH, whereas Na (EDX) was negatively associated with S. Cl (EDX) positively correlated with Na but negatively with S.

PCA explained 57.8% of the total variance (PC1 = 37.6%, PC2 = 20.2%) and partitioned traits into four major clusters (Fig. 7B): (i) Ca and Mg (AAS) grouped with pigments, proline, and flavonoids; (ii) oxygen and PheAce; (iii) Na, Cl, C, Mg (EDX) with ethanol-derived phenolics; (iv) Ca and S (EDX). Traits with loadings <0.5 were retained for completeness but were not emphasized. Overall, the ordination highlights a clear divergence between Ca-associated protective traits and Na^+^/Cl^−^-associated stress markers.

## Discussion

### Calcium uptake and biomineralization

The frequent occurrence of CaOx crystals in idioblasts and around vascular tissues of *A. sarmentosa* suggests that biomineralization plays a functional role, rather than being solely a metabolic by-product. Calcium stabilizes membranes, mitigates Na^+^ toxicity, and acts as a secondary messenger in stress signaling (Hadi & Karimi, 2012). Accordingly, CaOx crystals have been proposed to function as dynamic deposits linked to stress adaptation, carbon–calcium cycling, and, in some species, photosynthetic buffering through “alarm photosynthesis” (Tooulakou et al., 2016; Cuadra & Cambi, 2017; Gómez-Espinoza et al., 2020). In this framework, alarm photosynthesis is proposed to operate under stress-induced CO_2_ limitation (*e.g.*, salinity-associated stomatal restriction), and is discussed here as a conceptual physiological mechanism rather than a directly measured process. Because oxalate originates from CO_2_ fixation intermediates, CaOx deposition may contribute to the detoxification of excess Ca^2^^+^ and the temporary sequestration of CO_2_-derived carbon. Upon microbial degradation, these crystals could potentially release Ca^2^^+^ and CO_2_ back into biogeochemical cycles, highlighting a possible link between plant-level ionic regulation and broader carbon fluxes.

Comparative evidence supports the adaptive significance of CaOx-mediated biomineralization. In the halophyte *Sarcocornia pruinosa*, CaOx accumulation is enhanced under estuarine salinity (De la Fuente et al., 2018), suggesting a broader ecological relevance in salt-stressed environments. The diversity of CaOx morphologies (*e.g.*, druses, raphides) and their frequent confinement to idioblasts are associated with multifunctional roles in ionic sequestration, tissue-level defense, and structural support (Volk et al., 2002; Coté, 2009; Paiva, 2019). Importantly, CaOx formation is not exclusive to halophytes but represents a widespread plant trait, whose functional significance depends on ecological context and environmental constraints. In *A. sarmentosa*, the widespread deposition of CaOx across stems and leaves indicates a systemic strategy of internal calcium immobilization, reinforcing a calcium-centered form of salt tolerance.

### Salt glands and ion excretion

SEM imaging revealed bicellular salt glands on both adaxial and abaxial leaf surfaces of *A. sarmentosa*, consistent with the capacity for active excretion of Na^+^ and Cl^−^. Comparable structures are well documented in mangrove halophytes such as *Avicennia officinalis*, where secretion is dynamically regulated by aquaporins and ATP-dependent transporters (Tan et al., 2013). Although salt glands are not universally present across halophytes (Shabala, 2013), their occurrence in *A. sarmentosa* likely represents an external mechanism of ion removal that complements internal detoxification *via* CaOx biomineralization.

The coexistence of these two mechanisms—external salt secretion and internal ionic immobilization—underscores a highly integrative tolerance strategy. Similar multifunctional roles have been reported for CaOx crystals in other woody species, where phloem-associated deposits stabilize ionic balance and deter herbivory (Hudgins, Krekling & Franceschi, 2003). In *A. sarmentosa*, the combined action of salt glands and CaOx crystals may facilitate both rapid elimination of excess ions and longer-term immobilization within tissues, supporting survival in highly saline–sodic soils, particularly under conditions of limited calcium availability.

### Antioxidant and osmoprotective defense

Overall, young leaves of *A. sarmentosa* were the primary sites of osmotic adjustment and antioxidant defense. These findings are consistent with broader evidence that halophyte-derived metabolites, including phenolic acids and flavonoids, contribute to salinity tolerance by functioning both as antioxidants and signaling molecules in halophytes generally (Székely & Barta, 2025). Comparable patterns reported for *A. sarmentosa* populations from Thai salt-affected soils are consistent with the capacity of this species to mount enhanced antioxidant-related and osmoprotective responses under salinity stress (Poodeetip et al., 2013). More broadly, halophytes commonly employ osmoprotectants such as proline and trehalose to mitigate NaCl-induced oxidative damage (Liu, Jiang & Yuan, 2024; Manasathien & Khanema, 2024; Islam et al., 2025). Exogenous application of selenium and boron has also been shown in other plant systems to enhance antioxidant defenses and secondary metabolite accumulation under salinity stress, underscoring the role of mineral nutrients in maintaining redox balance and stress tolerance (Camlica, 2025).

Halophytes also deploy structural and molecular adjustments in their photosynthetic apparatus that interact with antioxidant defenses. Modifications in chloroplast ultrastructure and enhanced membrane lipid unsaturation help maintain photosynthetic activity under salinity (Rozentsvet et al., 2018). In *Zygophyllum propinquum*, salt exposure led to declines in *F*_v_/*F*_m_ and increased non-photochemical quenching, together with enhanced antioxidant activity, exemplifying photoprotective responses that may be relevant to *A. sarmentosa* (Gul et al., 2024). These observations are consistent with a coordinated accumulation of photosynthetic pigments, proline, and Ca in *A. sarmentosa*, suggesting a functional link between Ca-mediated ion regulation, photoprotection, and antioxidant buffering.

Enhanced antioxidant-related responses in young leaves of *A. sarmentosa* not only strengthen ROS detoxification but also reflect a broader stress response. Salt stress is known to induce flavonoid biosynthesis in halophytes, thereby enhancing oxidative stress tolerance and metabolic resilience (Feng et al., 2023). Beyond their antioxidant functions, flavonoids and related phenolics have been shown in other halophytic systems to serve allelopathic roles, as halophyte-derived secondary metabolites are increasingly recognized as modulators of plant–environment interactions and resilience under salinity (Székely & Barta, 2025). Likewise, edible halophytes often exhibit high antioxidant activity linked to phenolics and flavonoids, underscoring their dual contribution to stress physiology and human nutrition (Sánchez-Gavilán & De la Fuente, 2024). Reviews further emphasize that halophytes provide a rich reservoir of nutraceutical compounds (Lopes et al., 2023; Machado et al., 2024), placing *A. sarmentosa* within a broader group of halophytic species of ecological importance and potential agricultural value.

### Anatomical strategies under salinity

Anatomical observations revealed abundant crystalline deposits in the periderm, cortex, xylem, and idioblasts, together with bicellular salt glands on both leaf surfaces. These features are consistent with a dual role of ion immobilization and external excretion in *A. sarmentosa*. Previous studies have shown that salinity commonly alters xylem structure and promotes sclereid deposition, thereby reinforcing tissues against ionic and osmotic stress (Dolatabadian, Modarres Sanavy & Ghanati, 2011; Nassar et al., 2020). The occurrence of CaOx in vascular-associated tissues of *A. sarmentosa* resembles patterns reported for *Betula ermanii*, where bark and phloem crystals intensify under volcanic salt stress (Kopanina et al., 2022), highlighting the adaptive value of Ca-based biominerals in stress-prone environments.

Beyond ionic immobilization, such anatomical traits contribute to long-term mechanical resilience. Crystals embedded within vascular and cortical tissues likely enhance tissue rigidity, while idioblast-localized CaOx provides localized defense. The co-occurrence of structural reinforcement and salt excretion thus supports the capacity of *A. sarmentosa* to withstand the chronic ionic imbalance of saline–sodic soils, enabling both immediate stress buffering and sustained survival under prolonged salinity.

### Integrative tolerance strategy and implications

Integrating anatomical, biochemical, and physiological evidence, *A. sarmentosa* appears to employ a calcium-centered tolerance strategy characterized by: (i) apparent preferential Ca accumulation in Na-dominated soils; (ii) CaOx biomineralization in idioblasts and vascular tissues; (iii) Na^+^ and Cl^−^ excretion through salt glands; and (iv) osmotic and antioxidant buffering mediated by proline and photosynthetic pigments, with additional contributions from phenolics and flavonoids reported in other halophytic systems. Unlike soluble sugars or starch, which primarily support osmotic adjustment and metabolic energy supply, oxalate accumulation enables calcium detoxification, long-term ionic immobilization, redox buffering, and potential carbon sequestration under chronic salinity stress. Together, these tiers converge on a Ca-driven protective axis that complements, rather than relies solely on, Na sequestration. This may distinguish *A. sarmentosa* from some halophytes that primarily depend on sodium regulation. For instance, *Prosopis strombulifera* tolerates high NaCl but is inhibited by Na_2_SO_4_, reflecting salt-specific responses (Reginato et al., 2019), whereas *A. sarmentosa* persists in saline–sodic soils by emphasizing calcium enrichment and biomineralization.

Broader comparisons highlight the applied significance of such adaptations. Multi-year cultivation of *Suaeda salsa* improves saline soil structure, demonstrating the potential of halophyte-based phytodesalination (Liu et al., 2024). Similarly, intercropping with halophytes can reduce soil salinity and enhance crop yield (Jurado et al., 2024; Santin et al., 2024). At the same time, halophyte strategies may present trade-offs: oxalate accumulation can pose nutritional risks when consumed excessively (Barkla, Farzana & Rose, 2024). In this context, *A. sarmentosa* exemplifies a distinctive Ca-centered strategy with relevance not only for ecological survival but also for saline-soil management and the design of diversified agroecosystems, particularly in Ca-poor, Na-dominated landscapes where woody plant establishment is often constrained.

## Conclusions

*Azima sarmentosa* withstands salinity through a calcium-centered tolerance strategy that distinguishes it from halophytes relying primarily on sodium sequestration. In saline–sodic soils dominated by exchangeable Na^+^ and depleted in Ca^2^^+^, the species selectively accumulates calcium, stabilizes cellular structures, forms CaOx biominerals, and expels excess Na^+^ and Cl^−^ through bicellular salt glands. Young leaves, enriched in proline, phenolics, and flavonoids, further reinforce osmoprotectant and antioxidant defenses.

Multivariate analyses revealed a consistent divergence in trait associations: Ca-related variables clustered with pigments, proline, and flavonoids, whereas Na^+^/Cl^−^ grouped with crystal-associated elements and ethanol-derived phenolics. This pattern underscores the functional distinction between Ca-driven protection and Na-induced stress responses.

CaOx formation not only immobilizes excess Ca^2^^+^ but also incorporates CO_2_-derived oxalate, functioning as a transient carbon sink. This dual role links ionic regulation with carbon cycling, broadening the ecological significance of calcium-mediated salt tolerance. Together, these anatomical, physiological, and biochemical adaptations highlight *A. sarmentosa* as a resilient woody halophyte with potential relevance for managing saline–sodic soils and informing the development of salt-tolerant plant systems.

##  Supplemental Information

10.7717/peerj.21162/supp-1 Supplemental Information 1 Soil physicochemical properties The raw data used to generate Table 1. Measurements of exchangeable cations (K^+^, Na^+^, Ca^2+^, Mg^2+^), Cl^−^, SAR, EC_*e*_, pH, SOC, and TON from five sampling sites at two depths (0–50 cm and 51–100 cm).

10.7717/peerj.21162/supp-2 Supplemental Information 2 Ion accumulation and distribution in *Azima sarmentosa* Total and organ-specific concentrations of K, Na, Ca, and Mg, as well as calculated ionic ratios (K^+^/Na^+^ and Ca ^2+^ /Na^+^).

10.7717/peerj.21162/supp-3 Supplemental Information 3 SEM–EDX elemental composition of stems and leaves Normalized mass concentration (% weight) of elemental composition (C, Ca, Cl, K, Mg, Na, O, S) obtained from SEM–EDX analyses.

10.7717/peerj.21162/supp-4 Supplemental Information 4 Photosynthetic pigments Quantitative data for chlorophyll a, chlorophyll b, and total carotenoids in *A. sarmentosa* leaves.

10.7717/peerj.21162/supp-5 Supplemental Information 5 Osmoprotectants Quantitative data for proline and total soluble sugars in stems, mature leaves, and young leaves of *A. sarmentosa*.

10.7717/peerj.21162/supp-6 Supplemental Information 6 Antioxidant compounds Quantitative data for total phenolics and total flavonoids in stems, mature leaves, and young leaves of *A. sarmentosa*.

10.7717/peerj.21162/supp-7 Supplemental Information 7 Correlation matrix and principal component analysis (PCA) Raw data of all quantitative variables (AAS, EDX, pigments, osmoprotectants, and antioxidants) used to perform correlation and PCA analyses.

## Additional Information and Declarations

### Data Availability

The following information was supplied regarding data availability: The raw data is available at FigShare: Khanema, Piyanut; Laojinda, Woraporn; Rojviriya, Catleya; Manasathien, Jinnawat (2025). Comprehensive raw data supporting the manuscript “Calcium Dominance, Ion Regulation, and Metabolic Defenses Underlie Salt Tolerance in the Halophyte *Azima sarmentosa*”. figshare. Dataset. https://doi.org/10.6084/m9.figshare.30327991.v1.
